# Experience of individuals with unconsummated marriage in Iran: A qualitative content analysis

**DOI:** 10.18502/ijrm.v21i9.14401

**Published:** 2023-10-30

**Authors:** Sona Setayesh, Amir Hossein Jalali Nadoushan, Razieh Bakhshandeh, Reza Negarandeh, Mehrdad Eftekhar Ardebili

**Affiliations:** ^1^Mental Health Research Center, Department of Psychiatry, School of Medicine, Iran University of Medical Sciences, Tehran, Iran.; ^2^Mental Health Research Center, Psychosocial Health Research Institute, Department of Psychiatry, School of Medicine, Iran University of Medical Sciences, Tehran, Iran.; ^3^Nursing and Midwifery Care Research Center, Tehran University of Medical Sciences, Tehran, Iran.

**Keywords:** Consummation of marriage, Iran, Sex, Marital sex, Marital relationship.

## Abstract

**Background:**

Sex is one of the important aspects of marriage and a way of expressing intimacy. Unconsummated marriage is a common problem whose prevalence is about 1.5% in Iran and can significantly influence marital life.

**Objective:**

This study aimed to assess the unconsummated marriage in a qualitative mode to offer new and beneficial solutions resulting from a better understanding.

**Materials and Methods:**

In this qualitative study, conventional content analysis was used. Data collection and analysis were done from April 2013 to April 2014 with 11 women and 5 men participating, who were referred to the sexual disorders clinic of Tehran Psychiatric Institute and Rasool Akram hospital of Tehran, Iran. The researchers examined the files of people referred to these clinics with the diagnosis of unconsummated marriage. Our tool for data collection was a semi-structured interview. 2 experienced faculty psychiatrists recorded all the information based on standards for reporting qualitative research guidelines. Data were analyzed using qualitative technique and coding. Guba and Lincoln criteria was used for data validity.

**Results:**

The codes of the participants' experiences were classified into 6 main categories, which include self-concept, intrapersonal factors, partner's problem, preparedness for sex, emotional relationship between couples, and the effects of unconsummated marriage on the participant's life.

**Conclusion:**

Significant experiences among women included excessive anxiety toward the society and their spouses, and among men were weakness and guilt. Being unable to have sex negatively affected their view of life and caused great harm to their self-perception. Participants personality traits and their self-concept, and attitude toward their partners were important issues.

## 1. Introduction

People get married for different reasons. Sex, love, having a family or child, feeling protected, etc. A successful marriage could fulfill many psychological and physical needs in a safe environment and greatly influence the couple's mental health (1, 2). Sex is one of the essential aspects of marriage and an important way of expressing intimacy (3, 4).

Unconsummated marriage is a failure in vaginal intercourse attempts (5). It is a common problem that physicians face in some developing societies, which is the reason for almost 17% of sex clinic referrals (5-7). In Iran, the prevalence is estimated to be 1.5% (8). Various factors are involved in an unconsummated marriage, like anxiety, premature ejaculation, impotence, and vaginismus (9). Also, social and cultural factors are important in this problem. Education in sexual fields is essential, too (1, 10, 11). Pressure perceived by family and society for consummation on the wedding night is another crucial factor in increasing stress in couples. Besides, insufficient education is another stressor for a new bride and groom (12). The negative attitude of women toward their husbands is critical, too (13). The longer the problem lasts, the more difficult it is to treat (14).

There is insufficient research about unconsummated marriage, and available studies are in the quantitative method (15). Because of the importance of sexual satisfaction in the marital relationship and its negative consequences and for a deeper comprehension of this phenomenon, this qualitative study aimed to offer new and beneficial solutions resulting from a better understanding of unconsummated marriage.

## 2. Materials and Methods

In this qualitative study, data collection and analysis were done during the spring of 2013-2014 with the participation of 11 women and 5 men who were referred to the sexual disorders clinic of Tehran Psychiatric Institute, and Rasool Akram hospital, Tehran, Iran.

The method we used for this study is conventional content analysis. Content analysis is a way of having an objective and a common understanding of a text's verbal characteristics, and to deduce an author's personal and social characteristics, ideas, and attitudes. In qualitative research methodology, we do not have a predictable plan for case selection, data collection, and analysis (11). We have flexibility so that the research begins with an open question and goes on. Information collected from the first case guides us in selecting the next one.

### Participants

The number of cases was not determined before the termination of the research, and it was dependent on data saturation in this study. Data saturation refers to the point at which the new data being collected no longer provides any significant or new information related to the research question or objectives (11).

The inclusion criteria were that participants must have been at least 15 yr old and Iranian. People who are part of an unconsummated marriage, that is, people who have not had sex even though they are married, even after a day of marriage. Individuals voluntarily and willingly agree to participate and should be open to discussing their experiences, feelings, and challenges related to their unconsummated marriage.

The exclusion criteria were having diagnosed severe mental disorders such as psychotic disorders, using medication that would affect sexual desire such as selective serotonin reuptake drugs, sedative-hypnotic drugs such as benzodiazepines, using antipsychotics drugs, chemotherapy, and anti-cancer drugs, using estrogen and progesterone, anti-acne drugs, cardiovascular drugs, and antihypertensive drugs, being addicted to drugs and alcohol, having diagnosed with untreated diseases such as diabetes and hypothyroidism, and having a severe restrictive physical activity that interferes with having sex, and participants who do not want to participate in the study.

### Procedure

The duration of the research was 9 months. The researchers examined the files of people referred to these clinics with the diagnosis of unconsummated marriage, called them, and invited them to the clinic, then in a 10-min interview with a participant to explain the study format. 2 experienced faculty psychiatrists conducted the interviews for 45 min for each participant. The corresponding author supervised all the consultations, data-gathering processes, and categorizing stages.

Our tool for data collection was a semi-structured interview. First identifying data, including name, age, education, occupation, duration of marriage, and psychiatric diagnosis. We started interviewing with an open question: “What is your feeling about your problem”? According to the participant's answer, the following questions were determined, such as:

“What is the effect of your problem on your marital relationship?”

“What is the effect of the problem on your mood”?

“How is your attitude towards sex”?

“How was your last experience in sex with your husband”?

“How do you know your husband”?

The researcher recorded all the information and nonverbal gestures and postures that participants expressed during their interviews. A qualitative study report checklist was prepared based on standards for reporting qualitative research guidelines (15).

The interviews were immediately written and implemented and sorted for qualitative content analysis. Then the whole text was read several times to get a complete familiarity with the data before starting the coding.

Semantic units and primary codes were extracted from the raw data. At this stage, stabilization in coding was achieved.

In open coding, the data was examined line by line and a code was assigned to each related sentence or event, and identified. After coding the whole text, consistency in coding was checked again.

In the next step, the primary codes were classified, and similar codes were placed in categories. Categories were extracted from the raw data with an inductive approach. The researcher found the distinction between the categories by using the method of continuous comparison of ability. A summary table of codes and categories was prepared. The quotes that represented each of the classes were placed on the table and completed in the same way until the last interview. Codes with similar concepts were grouped together to form categories. New codes were added to the initial table and were placed inside the categories. The codes were progressively reduced over time, and this process ended with the analysis of the last manuscript.

In this study, we used Guba and Lincoln criteria for data validity. This criterion consists of transferability, confirmability, trustworthiness, and credibility (16).

Credibility is a valuable and common device for the truth and accuracy of results and data. Guba and Lincoln describe this so that the study process is coordinated with facts of study data. To reach this purpose, long-term engagement with data, combining study methods and data resources, analysis of cases, and data confirmation by participants is necessary.

To gain trustworthiness and reliability, all study processes were recorded accurately. In addition to transferability, which means that people other than participants with the same problem studying results and data feel a resemblance between them and their experience. The qualitative researcher checked the results with 2 other patients with unconsummated marriages, and they perceived effects the same as they experienced. For confirmability, the researcher recorded the study process for follow-up and more investigation in the future. To increase the accuracy of results, a qualitative researcher reviewed codes and checked them with texts.

### Ethical considerations

This study was approved by the Ethics and Research Committee of Iran University of Medical Sciences, Tehran, Iran (Code: IUMS.FMD.REC.1391.333). Written informed consent was obtained from all participants to conduct an interview and participate in the study. After the interview, the participants were offered free treatment and counseling sessions. Confidentiality was explained and promised to participants. We got their agreement to record their voice and reassured them to delete that after registration.

### Statistical analysis

After completing each interview, the researcher wrote the text of the conversations using the (listen and write) software, the interview was written verbatim and then coded. After the analysis of each interview, the next interview was conducted. Information was analyzed via MAXQDA software (professional software for mixed methods and qualitative data analysis). In the conventional content analysis method, codes and classes are directly and inductively extracted from raw data. Data analysis were done with the approach of contractual content analysis and their processing with the systematic method of Graneheim and Lundman (17).

## 3. Results

11 women and 5 men participated in this study. Table I shows the demographic characteristics of the participants.

In total, 1100 conceptual codes were extracted, divided into 2 general categories of women and men's experiences with 6 main categories. The main categories and examples of main codes are given in tables II and III.

### Self-concept 

#### Women

The self-concept means self-recognizing or understanding a person about herself and how she perceives her partner and marital life. In this category, we had these codes: a sense of being unlovable, being over-controlled, not being a good wife, risk-taking and assertiveness, dependence, and fear of intimacy with a man. One of these codes was a sense of responsibility toward the partner, it results from self-blaming. She thought that she was responsible for the sexual problem and that her husband was entirely healthy. Self-blaming and a sense of responsibility result in guilt and self-pitying.

#### Men

Compared to women, male participants talked less and were less willing to visit, and more women sought treatment. Codes such as low courage, being emotional, good social and professional relationships, not being afraid of having sex with someone else, a sense of fear and good self-confidence, and not being strict were obtained.

One of the men considered himself to be a timid person. Besides these qualities, he felt very emotional and romantic, and he saw these tender feelings of himself being incompatible with his wife. He considered himself unsuccessful in sex and felt quite successful in career fields. He thought that there would be no problem if he had sex with someone else and mentioned that he had sex before marriage and there was no problem.

Most of the participating men did not feel weak and weak about themselves; on the contrary, they thought that their women had problems and did not have any issues, and there was no need to do anything for them.

### Intrapersonal factors 

#### Women

Anxiety is a feeling that participants experienced before sex and felt that it inhibits sexual arousal and excitement. They experienced low self-esteem and a sense of weakness with anxiety. One of the participants said:

“I contract my body and scream and feel severe anxiety and fear for penetration of the penis into my urethra or anus". She experienced anxiety with somatic symptoms such as muscle contraction, tension, and palpitation. Fear of being ruptured and traumatized leads to anxiety. One of the women believed that her vagina was too small to be penetrated by her husband's penis. Anxiety as a personality trait was known in some participants. One of the participants had experienced vaginal trauma in her childhood, and she always feared being traumatized even while washing her external genitalia. Anxiety also can be a consequence of unconsummated marriage. This feeling is reported to result from the sense of unsuccessfulness in consummation. Anxiety is composed of various forms of fear, such as fear of pregnancy, inadequacy, being injured and ruptured, fear of a too-large penis, fear of being utterly passive towards her partner, acceptance of someone else's authority, fear of bleeding, etc. Inhibition for penetration was a typical result of all these fears. Another etiological factor in evoking anxiety was the sense of obligation for consummation on a wedding night or a few days after.

#### Men

In the sub-category of fear and anxiety, which consisted of different worries, such as fear of blood and bleeding in women, fear of injections, fear of first intercourse, and fear of intimacy, the participant felt all these fears in a short time during intercourse, and these prevented his ability to penetrate.

In the perception section, the participants mentioned their opinions about the child. For some, the existence of children is fundamental to them, and some value sex more than the presence of children to solve problems, and some do not want to have children at all. One of the participants said that sex had become a common issue for his wife, but it would never be normal for him.

Most of the men mentioned the feeling of not being able to have sex, and at this time, they were upset and disappointed, and it was difficult for them to express such a feeling. Most of them wanted their wife's virginity to be lost somehow, and they even preferred it to be done by a surgical blade so that their trouble would be less. One of the participants wanted sex to be removed so that he could live with his wife without sex.

In the experience of sex with their spouse, they found flirting before intercourse to be very effective in stimulating enough for sex, and they described it as low.

One of the participants said, “he is going to sleep with a dead body, and this leads to a feeling of death and coldness in his body. He felt that his wife is an insensitive woman, and she is not stimulating to him at all, and she has no sexual desire for him”.

Men's recognition of their women included several codes. Most of them felt that their spouse was running away from the problem and avoiding sex. Those who considered themselves guilty had the feeling that their spouse was inclined to divorce and wants to separate.

One of the men said that according to his wife, he is very small and weak and very positive, and he does not know how to have sex, and his wife tells him to go to the gym to become more masculine. Most of the men felt that their wife were not interested in hymenectomy and it is considered painful for them.

### Partner's problem

#### Women

Another concept was the attitude of women toward their partners. The man's personality wishes for having a child, desire for sex, attitude toward treatment, history of sexual abuse in man, sense of rape to his wife during sex for man, fetishism and voyeurism in man, fear of bleeding and blood, and injection in man, man's fear of driving and sexual immaturity in man can all affect women's attitude.

Willing to have a child was a determination to try enough and motivate for treatment. Another concept was fear in man. Participants reported fear in their husbands, like fear of blood and woman's bleeding, fear of driving, fear expressed as performance anxiety and social phobia. One participant reported a history of oral sexual abuse in her husband in his adolescence. He re-experienced the trauma during intercourse, which resulted in low sexual desire.

One concept in this category was the sexual and emotional intimacy between the bride and groom's parents. They thought that an intimate relationship between their parents could be a good factor for their sexual maturity and vice versa. Some participants felt they did not have sexual desire for their husbands and could not enjoy sexual contact. One of them perceived her husband as not physically attractive and not masculine enough to be aroused by him.

Most of the participants reported the insistence of their partners for hymenectomy. Still, they were reluctant to do it and preferred to experience consummation by their husband, not by a surgical or artificial procedure.

Another concept was activity and passivity in sex. One of the participants liked to feel active during intercourse and sometimes be passive toward her husband and be under his control in sexual activity. She said, “when my husband thinks that I have pain during intercourse, he gives up, but he does not know that the pain of being unconsummated is more than the pain of penetration”.

#### Men

Some male participants mentioned that their partners do not understand them and that there is no love or sex in their lives. One said, “maybe our behavior is wrong, and the type of our request is wrong”. Some people have proposed prolonging the marriage period and the absence of sex during this period, causing problems. Most of them were interested in their spouses and were satisfied to continue living with them even without sex and could not bear to be separated from their spouses. One of the participants said that he does not talk about the type of relationship with his wife and puts all the critical issues of life in a box and closes the door.

### Preparation 

#### Women

Another concept was preparedness which consists of previous sexual activities and sexual education. Romantic relationships before marriage, history of vaginal trauma, and aversion to sex were necessary codes in this category. Participants thought that having sexual and emotional relationships before marriage could be a good factor in more sexual maturity. Not having these relationships can be a determinant in creating that problem.

Education in sexual issues was an important category that indicates social education and education by parents and social and family attitudes toward sex. Participants stated that education about sexual matters is insufficient in our society, and it is taboo. It is not common for the Iranian families to discuss any sexual-related topic with their children, as a result, there is a high likelihood for the children to face challenges regarding such topics and activities. For example, one of the participants expressed she did not know she has not experienced penetration and complete intercourse for a long time, she and her husband thought they had intercourse, and during a conversation with their friends, they understood their problem.

#### Men

A person's previous experiences with sex were a factor in favor of solving the problem, and not having it means that the person is more problematic. In some participants, insufficient sexual knowledge has been an influential factor because, for a long time, they did not know that their relationship with their wives was not perfect sex at all. He considered society and family to be very involved in this matter and said that in Iran, they cover up sexual issues so that there is no discussion.

### The emotional relationship between the couple

#### Women

Another category was the emotional relationship between couples. Not enough emotional support awakens a sense of inferiority, insufficiency, and low self-esteem. It can worsen the problem. Romantic attachment between couples is an essential factor independent of sex. One of the participants stated that despite their sexual issues, they love each other more than before. In some participants, insufficient emotional support resulted in low sexual desire. Some participants felt that being supported by their husbands is a protective factor. One of them was satisfied by only foreplay and without intercourse in man. The inability of a man to penetrate was another concept expressed in various forms, such as premature ejaculation, fear of penetration, fear in women and inhibition against penetration, insufficient excitement, and inadequate arousal. Participants felt that when they find their partner not sexually attractive and active and not aroused enough, they lose their excitement and feel angry about their partner because they find them fearful and weak.

#### Men

Some men feel more comfortable expressing emotions openly, while others struggle due to societal expectations or personal beliefs. One of the participants said that “being able to be vulnerable with a partner is essential for building trust and deepening emotional bonds”. Emotional intelligence, which involves recognizing, understanding, and managing emotions, can impact their ability to navigate emotional relationships. Some men have more direct approach, while others may prefer to avoid conflicts. Traditional gender roles or societal views on masculinity, may shape men's attitudes toward emotional expression and intimacy.

### Effects of unconsummated marriage on participant's life

#### Women

In this category effects of this problem are further divided into 3 subcategories, effects on participant's feelings, effects on marital relations, and effects on participant's behavior or behavioral changes. The first subcategory reported the feelings such as sadness, negative feelings, and depression, leading to functional impairment. Sense of inability and inefficiency, and low self-esteem were other effects of this problem. Some participants thought they could not do a function that even animals can, so they felt that they had a significant defect because of not having sex. Some participants feared divorce and were nervous about their husband's extramarital sexual contact, which resulted in more anxiety and led to behaviors such as being utterly passive toward their partner and limiting their freedom.

Some of them thought their partner had sexual dysfunction and felt they could not have regular sex because of their husband's dysfunction, which resulted in aggression toward their partners. Another feeling was the sense of inferiority. This problem led to behavioral changes, such as social isolation and limiting familial relations. Women felt additional pressure from their family members. Their parents insisted on childbearing, and so they had to isolate themselves.

#### Men

The feeling of weakness was present in all the men who saw themselves as guilty and those who wanted thymectomy in their wives; this preference led to a sense of lack of zeal in the individual.

The feeling of sadness and discomfort from the inability to have sex was a familiar feeling in most of them, and in one person, it had progressed to the point of suicidal thoughts.

One of the participants saw himself in this predicament of living alone so that his wife would be out of the house for long periods for her entertainment, lest she leaves him.

There was a feeling of disappointment in several of them, and it was because of hearing about the scandal from their wives and the people around them. The effects of this problem on the marital relationship included separation and coldness of the spouse and arguments. The degree of sense of ability and powerlessness was related to feeling guilty.

**Table 1:** Demographic characteristics of male and female participants


**Participant**	**Age (yr)**	**Education**	**Job**	**Duration of marriage**	**Psychiatric diagnosis**
**F1**	27	B.Sc. of Psychology	Psychologist	4 yr	None
**F2**	26	B.Sc. of Law	Housewife	4 yr	None
**F3**	26	GP	Medical doctor	1 yr and 9 months	GPPPD
**F4**	26	M.Sc.	Employed	4 yr	None
**F5**	21	Diploma	Housewife	2.5 yr	None
**F6**	31	B.Sc.	Kindergarten teacher	5 yr	None
**F7**	20	Medical student	Student	2 wk	GPPPD
**F8**	30	B.Sc.	Company's employee	10 months	GPPPD
**F9**	30	Diploma	Housewife	2.5 yr	None
**F10**	27	B.Sc.	The coach	3 months	None
**F11**	26	Associate degree	Company secretary	1 yr	None
**M1**	34	M.Sc.	Employed	4 yr	Premature ejaculation
**M2**	34	M.Sc.	The architect	4 yr	None
**M3**	32	M.Sc.	Employed	4 yr	Male hypoactive sexual disorder
**M4**	26	Diploma	Shopkeeper	2.5 yr	Voyeuristic disorder/fetishistic disorder
**M5**	31	B.Sc.	Bank employee	5 yr	None
F: Female, M: Male, B.Sc.: Bachelor's degree, M.Sc.: Master's Degree, GP: General practitioner, GPPPD: Genito-pelvic pain/penetration disorder					

**Table 2:** Main category and example of main codes in female participants


**Main category**	**Example of main codes**
**Category 1:** **Self-concept**	Bad body image, a small view of the vagina, a sense of complete empowerment in a woman, feeling anxious, the idea of being hot, being sensitive
**Category 2:** **Intrapersonal factors**	Prevent penetration, fear of disability, fear of pregnancy, fear of damage and tearing,
fear of pain, fear of bleeding;
Shame on getting naked in front of the husband
Lack of desire for the wife; dissatisfaction and lack of sexual pleasure;
difficulty of hymenectomy
The desire to dominate the man; the importance of a large and masculine body;
do not want children; interest in children
Low effort; great effort; inability to penetrate; premature coldness during sex
**Category 3:** **Partner's problems**	Premature ejaculation; a positive man; childhood man; skinny man; gentle man;
a man with feelings; fear of penetration and squeezing and damage
**Category 4:** **Preparation**	Lack of love and sex before marriage, recalling the memory of rape during sex with a partner, rape of a man in adolescence, aversion to sex Not knowing about sex, lack of education by mother, lack of education in society, sex is taboo in society
**Category 5:** **Emotional relationship**	Lack of love from husband, getting a sense of low value from a man, expressing feelings of inadequacy of the man. Not understanding my hints
**Category 6: ** **Effect on life**	Feeling depressed, feeling weak, disappointed, self-blame and guilty conscience, anxiety,
decreased desire for a spouse, feeling sick
Respecting the husband; expressing a decrease in desire for a spouse; isolation; divorce
legal action;
Couples moving away from each other; reluctance in man, discomfort in man, partner
getting angry

**Table 3:** Main category and example of main codes in male participants


**Main category**	**Example of main codes**
**Category 1:** **Self-concept**	The feeling of not being liked and bad self-image, bad body image, a small view of the vagina, a sense of complete empowerment in a woman, feeling anxious, the idea of being hot, being sensitive
**Category 2:** **Intrapersonal factors**	Prevent penetration, fear of disability, fear of pregnancy, fear of damage and tearing,
fear of pain, fear of bleeding,
Shame on getting naked in front of the husband
The sexual and emotional closeness of my parents and the reverse of this issue
in the case of my husband,
Mandatory sex on the wedding night; lack of desire for the wife; dissatisfaction and
lack of sexual pleasure;
Desire to continue sex despite the pain, the importance of a large and masculine body;
Inability to penetrate, premature coldness during sex and being sensitive
**Category 3:** **Partner's problems**	Premature ejaculation, a man's satisfaction by just making love; full physical health of the man; a positive man, childish man, skinny man, gentle man, fear of penetration and squeezing and damage
**Category 4:** **Preparation**	Lack of love and sex before marriage, recalling the memory of rape during sex
with a partner, rape of a man in adolescence, aversion to sex
Not knowing about sex, lack of education by mother
**Category 5:** **Emotional relationship**	Lack of love from husband; getting a sense of low value from a man; expressing feelings of inadequacy of the man; not understanding my hints
**Category 6:** **Effect on life**	Feeling depressed, feeling weak, disappointment, self-blame and guilty conscience,
anxiety, decreased desire for a spouse, virginity, sense of inferiority
Respecting the husband; expressing a decrease in desire for a spouse; dowry forgiveness;
divorce legal action
Couples moving away from each other; reluctance in man

## 4. Discussion

The results in this research showed that women and men with unconsummated marriages experience various feelings. Their self-concept, personality traits, and their attitude toward sex have a significant effect on their sexual life and marital relation. Other researchers confirm these results and state that predisposing factors in this problem include temperament and constitutional elements, attachment issues, parenting, and a history of neglect and abuse (9, 18). These factors result in more sexual and emotional problems in adulthood (11). Factors such as problematic relationships, performance anxiety, guilt, inadequate sexual information, inadequate foreplay, mental illness, fear of intimacy, low self-esteem and distorted self-image, and non-intimate marital relation can perpetuate unconsummated marriage in couples (2, 11).

Fear of penetration because of pain; fear of bleeding, physical damage, and injury; and fear of pregnancy contribute to sexual inhibition, and participants sense fear in muscular and vaginal constriction forms. In some cases, women avoid sex, which leads to this problem (7). The conception of the perforation of the hymen to be associated with pain and bleeding leads to fear in women. These fears in women result in anxiety, and participants experience severe anxiety during intercourse related to bodily symptoms such as palpitation and tension (6). Anxiety as a personality trait can predispose women to multiple fears. Research confirms the role of anxiety in unconsummated marriage (19). In men, the fear of not being able to penetrate, the fear of bleeding in a woman, the anxiety of seeing blood, and other worries, such as the fear of hurting a woman and squeezing and penetrating a man, made them unable to do this. In one of the participants, these fears caused him to sweat and tremble during sex; in addition, he saw his wife so little in expressing emotions that he felt he was going to have sex with a corpse, and this caused him double fear. Another reason for impotence in male participants was premature ejaculation, which has been discussed in other studies (5, 17, 18). According to Freud's theory, most sexual disorders originate from the oedipal period, and the fear of sex may result from insincere childhood communication (14). The female partner's role is vital in treating and creating sexual dysfunction. The female partner is an essential factor in stimulating the male in sex. A woman's satisfaction affects a man's sexual behavior (20).

Participant's attitude toward a partner has a significant role in unconsummated marriage. When a woman knows her partner fears assuming she is more sexually mature, she does not allow her partner penetration. The unloving emotional relationship between a couple can result in insufficient sexual tendency. Marital conflicts, besides aggression and anxiety, can reduce sexual desire (19, 21). Studies explain vaginismus as a symbol of hostility and defense of a woman against her partner's sexuality (7, 9, 22). This conception that sex is for men's enjoyment and only for sexual satisfaction results in more aggression and inhibition. Cognitive dimensions in interpersonal and marital relationships are also critical (23, 24). As for beliefs about sexuality, the existence of conservative ideas about sexual behavior, for example, masturbation, oral sex, and anal sex, are considered deviant and sinful, sex is a male activity, and women need to control their sexual drives and pleasure because it has wicked experiences (25). In men's attitude, the type of emotional relationship between husband and wife has a significant effect on sexual relations, and if a woman is erotic and attractive to men, it can effectively reduce sexual problems, or on the contrary, it makes sex not happen ultimately.

Women with unconsummated marriages isolate themselves and hide their problems and consequently do not come for therapy or come very late (23). They discontinued their relationship with their friends who had children, and their coping mechanism was avoidance. This problem has a wide range of adverse effects on marital relations (22). Some of them felt that they were responsible for their problem, and this feeling resulted in self-blaming and guilt. So, they changed their behavior in favor of their partner's wishes. Hence, women lose their satisfaction in their lives, and their intimacy with their partner diminishes, and consequently, their sexual desire and satisfaction decrease. In this situation, participants had a divorce and were nervous that their partner would get extramarital sexual contact (24). Also, the feeling of depression was present in male participants, and the intensity of this feeling was related to the power of coldness in the emotional relationship between husband and wife. The more the man perceived the love between the couple and the greater the intimacy between them, the lower the level of depression. This feeling of sadness and discomfort among the male participants had external effects in the form of changes in performance and behavior, which led to frequent suicidal thoughts in one of the men, he considered himself entitled to forced solitude and giving complete freedom to his wife, and he did not feel any rights for himself and was desperate for treatment. The fear of being judged by others for not having children and the feeling of being left behind and being in the minority in the participants led to social anxiety in one of the participants. Men who felt defective in themselves were worried that their wives would want to divorce them. In this situation, he tried to be satisfied with sex without penetration and sexual fantasy, and in any way, he could only stay with his wife, and he even thought of removing sex from his marital life.

Another challenge that participants experienced was related to the relationship status mentally, in other words, the husbands didn't mentally see their wives in a partner role. In addition, spouses would only accept their virginity loss to occur with their partner and not with any external means such as surgical operations. Studies confirm that a person's feelings toward their partner substantially affect sexual interests and even hormone levels (18, 25). A woman who sees her husband as weak and imagines that he does not know how to communicate with her and sees herself as more dominant in sex cannot entrust herself to a worthless husband. Such a feeling in a man leads to a greater understanding of the feeling of weakness and deficiency, strengthens his inability to penetrate and his fears in sex, and has influence on the couple's emotional relationship. Such feelings have often led to anger and quarrels between the couple and divorce. Participants complained of low sexual education in families and society and stated that speaking about sexual issues is inhibited, an important factor in creating this problem. Studies confirm that even discussing sex in Iranian culture is taboo. Some authors have discussed the role of cultural determinants but in this study, the participants did not mention religious issues (26, 27). Parenting has a vital role in the growth of sexual information of children. Rigid and stubborn parents may suggest to their children that sex is dirty, resulting in sexual inhibition (28). Insufficient sexual education and religious and cultural limitations are important factors in this problem. So good and enough education can help people be protected from this kind of sexual dysfunction. Unconsummated marriage has various effects in a wide range on all life of participants. They feel sad and feel that they have a defect. Studies have paid enormous attention to the relationship between depression and sexual problems. Depression results from sexual dysfunction and can lead to sexual problems (29, 30).

## 5. Conclusion

We found that women and men with unconsummated marriages encountered psychosocial problems due to the inability of having sex, anxiety and depression were frequent problems that these individuals encountered. Significant experiences among women included excessive anxiety toward the society and their spouses. And among men were weakness and guilt. Being unable to have sex so negatively affected their view of life and caused great harm to their self-perception. Personality traits in participants, their self-concept and attitude toward their partners were important issues. Giving information about sexual issues has a positive effect in reducing this phenomenon.

### Limitations

Due to cultural and social norms in Iran, it may be difficult to recruit participants who are willing to discuss their experiences with unconsummated marriages. This may limit the sample size and diversity of participants, which could affect the generalizability of the findings.

Researchers may face bias in data collection due to cultural and social norms that may lead respondents to provide socially desirable responses or conceal certain information. This may affect the accuracy and reliability of the research findings.

There is a lack of comparative data on unconsummated marriage in other countries or cultures, which may limit the ability of researchers to draw meaningful comparisons or identify cross-cultural patterns. Unconsummated marriage is a sensitive and taboo topic in Iranian society, and people may be reluctant to discuss it openly. This can make it difficult for researchers to find participants who are willing to share their experiences and perspectives.

Iranian culture places a high value on family and marriage, and there may be pressure on couples to maintain the appearance of a happy marriage, even if it is unconsummated. This can make it difficult for individuals to seek help or support from their families and communities.

Overall, these limitations highlight the need for careful consideration of the cultural and social context of unconsummated marriage.

##  Conflict of Interest

The authors declare that they have no competing interest.
